# Segmental Aging Underlies the Development of a Parkinson Phenotype in the AS/AGU Rat

**DOI:** 10.3390/cells5040038

**Published:** 2016-10-17

**Authors:** Sohair M. Khojah, Anthony P. Payne, Dagmara McGuinness, Paul G. Shiels

**Affiliations:** 1 School of Life Sciences, Pharmacology Research Theme, University of Glasgow, Glasgow G12 8QQ, UK; smkhojah@yahoo.com (S.M.K.); Anthony.Payne@glasgow.ac.uk (A.P.P.); 2 Wolfson Wohl Cancer Research Centre, Institute of Cancer Sciences, University of Glasgow, Glasgow G61 1QH, UK; Dagmara.McGuinness@glasgow.ac.uk

**Keywords:** AS/AGU rat, sirtuins, p16^Ink4a^, SA-β-Gal, senescence, brain, Parkinson’s disease

## Abstract

There is a paucity of information on the molecular biology of aging processes in the brain. We have used biomarkers of aging (SA β-Gal, p16^Ink4a^, Sirt5, Sirt6, and Sirt7) to demonstrate the presence of an accelerated aging phenotype across different brain regions in the AS/AGU rat, a spontaneous Parkinsonian mutant of PKCγ derived from a parental AS strain. P16^INK4a^ expression was significantly higher in AS/AGU animals compared to age-matched AS controls (*p <* 0.001) and displayed segmental expression across various brain regions. The age-related expression of sirtuins similarly showed differences between strains and between brain regions. Our data clearly show segmental aging processes within the rat brain, and that these are accelerated in the AS/AGU mutant. The accelerated aging, Parkinsonian phenotype, and disruption to dopamine signalling in the basal ganglia in AS/AGU rats, suggests that this rat strain represents a useful model for studies of development and progression of Parkinson’s disease in the context of biological aging and may offer unique mechanistic insights into the biology of aging.

## 1. Introduction

The AS/AGU (Albino Swiss/Anatomy Glasgow University) rat strain derives as a single gene mutant for protein kinase C gamma (PKCγ) from an Albino Swiss (AS) rat parental strain. It displays a largely neuronal phenotype associated with the disruption of neurotransmitter (dopamine and serotonin) release within the basal ganglia and consequently, this is accompanied by the development of a Parkinsonian phenotype, including a staggering gait, tendency to fall, bradykinesia, akinesia, and a whole-body tremor manifesting at an early age [1]. These manifest without any notable changes in the cardiac or pulmonary systems [2,3,4,5]. The reduction of neurotransmitters in the dorsal caudate putamen by the age of three months is concurrent with an overall reduction of whole tissue dopamine levels at six months, in comparison to the parental AS strain, and a reduction of tyrosine hydroxylase in the substantia nigra compacta (SNC) at 12 months [1,5,6]. A unique feature of the AS/AGU mutant is its associated motor dysfunction, a direct result of dopamine disruption, which can be reduced by l-dopamine administration, and which has been linked to differential glucose usage in the basal ganglia, with no observable gross morphological differences between the AS/AGU and AS strains [7]. In contrast to the AS/AGU strain, other PKCγ knockout mouse models present only mild motor impairment, related to the loss of ability to eliminate the innervations from Purkinje cell climbing fibres early in the post-natal period. However, in these models, the motor impairment is not linked to dopamine disruption, but to additional changes in long-term potentiation in the hippocampus [8,9]. However, a recent phosphoproteomic study into the regulation of dopamine release has identified PKCγ substrates, in particular Pak-interacting exchange factor-β (βPIX) phosphorylation, as a positive modulator of processes via the PKCγ-βPIX-Cdc42/Rac1 axis [10].

Given the difference in lifespan between the AS/AGU mutant and AS parental strain, due to the effects of a single gene, the AS/AGU rat provides a unique opportunity to determine if there are any differences in the rate of aging across different brain regions [1]. This is pertinent to a better understanding of inter-individual differences in aging and age-associated health over the life course and offers the direct potential to evaluate such changes in the context of whole-body aging [11,12,13]. Importantly, it offers a unique opportunity to evaluate whether there are segmental aging processes at work in the brain due to the loss of PKCγ, or whether the rate of aging is uniform in the whole organ. Critically, given the lack in functional differences in other major body systems (e.g., cardiac and pulmonary systems) between parent and mutant, a greater acceleration in aging across different regions of the brain in the same individual might, therefore, exemplify segmental or “decoupled” aging processes [12]. 

Studies on age-related changes in the brain have typically focused on the emergence of physiological dysfunction inherent in neurodegenerative diseases. These are often improperly classified as diseases of accelerated aging, measured by region- or cell-specific atrophy, increased cellular senescence, changes in the neuronal connections, or changes in particular brain region activity, accompanied by neurochemical changes related to neuronal and axonal loss [14,15,16,17]. However few studies have focused on aging in the healthy brain.

The aging process, and any associated differences in rates of aging, can be monitored using validated biomarkers of aging (BoAs) [13]. The use of validated BoAs can provide accurate reports on cellular metabolic status, cell replication capacity, DNA damage status, redox stress levels, and changes in the cell phenotype or secretory components. 

Recently, cyclin-dependent kinase inhibitor 2a (CDKN2A/p16^Ink4a^) expression has been identified as a superior BoA [13,18]. Biological aging and cellular growth arrest linked to CDKN2A expression are inherent to the intracellular trinity comprising mitochondrial activity, telomere function and ribosome biogenesis (MTR) [19]. This intercommunicating network facilitates cellular decision-making in response to stress. Its component parts allow the cell to identify the level of stress/damage and to determine how much fuel to burn and energy to expend to effect repair, enter senescence or undergo cell death. Key mediators and effectors of these cellular decisions are the members of the sirtuin family [20]. The brain has the highest energy requirement in the body in order to maintain its functionality, thus, the activity of sirtuin family members may provide a critical link between cellular metabolism, cellular damage responses and, hence, aging in the brain [21,22]. In support of such a hypothesis, Sirt5, in particular, has been reported to act as a neuroprotective agent against motor deficit and dopaminergic degeneration in the MPTP (1-methyl-4-phenyl-1,2,3,6-tetrahydropyridine)-induced mouse model of Parkinson’s disease [23]. Sirt6 also appears to act differentially in the brain in comparison to other organs. For example, Sirt6 full knock-outs die due to hypoglycaemia, whereas neuron-specific deletion of Sirt6 does not have the same effect. However, Sirt6 knockouts appear to demonstrate a progeric phenotype, inclusive of impaired telomere function, genomic instability and dysregulation of cellular metabolism [24,25,26,27]. Sirt7, although it is the least characterized of the sirtuin family members, has been implicated in the regulation of ribosomal biogenesis and protein synthesis, cell proliferation, maintenance of DNA integrity, and prevention of DNA damage [28], key features of cellular aging processes.

In this study we have sought to determine whether the AS/AGU rat strain displays features consistent with accelerated brain aging and if a segmental aging phenotype is apparent in relation to the parental AS strain. To achieve this, we have used a panel of markers of cellular aging, including senescence-associated β-galactosidase (SA-β-gal), and expression of Cdkn2a/p16^Ink4a^ and sirtuins 5, 6, and 7 (Sirt5, Sirt6, and Sirt7). The combination of these markers covers the functional interactions of the components of the MTR trinity. We have hypothesized that a molecular signature based around the MTR trinity may offer a new insight into basal ganglia disorders, and mechanistic insight into aging processes in the brain. Notably, in this study, we specifically chose to investigate only male rats to remove any additional effect from steroid hormones, particularly oestrogen, which has been shown to be protective against mitochondrial damage and oxidative damage overall by affecting both the nuclear genome and mitochondria [29].

## 2. Materials and Methods 

### 2.1. Tissue Collection and Processing

AS and AS/AGU rat strains used for these experiments were maintained in adherence with the local and national regulations. Both rat strains received a standard diet, with drinking water ad libitum. Animals were reared and housed in the Joint Research Facility, University of Glasgow (Glasgow G12 8QQ, UK) under standardized conditions: light/dark cycle, 12/12 h; temperature 22 ± 2 °C; and humidity 50% ± 5% in plastic-metal cages.

Overall, twenty-four male rats were sacrificed using carbon dioxide euthanasia, six for each of the four experimental groups were used; these included AS rats (two months and 12 months old) and AS/AGU rats (two months and 12 months old). Dissected whole brains were cut in half mid-sagittally with half of the tissue being snap-frozen and further processed for IHC staining ,while the remaining half was snap-frozen in liquid nitrogen and stored for further analyses.

Briefly, brain tissue collected for further immunohistochemical staining were snap-frozen in liquid nitrogen, embedded in Tissuetek OCT compound (OCT, Merck Ltd, Nottingham, UK), quickly frozen in isopentene cooled with liquid nitrogen, and cut into 10 µm tissue sections using standard procedures. These sections were fixed in 95% ethanol and stored for further analyses.

### 2.2. Immunohistochemistry

Fixed tissue sections were allowed to warm up to room temperature, rehydrated with phosphate-buffered saline (PBS, Gibco, Paisely, UK), and treated with 3% hydrogen peroxide (10 min, RT; Sigma-Aldrich, Dorset, UK) to remove endogenous peroxidase activity followed by 5 min wash in distilled water. 20% normal goat serum (#PCN5000; Invitrogen, Paisley, UK) in Tris-buffered saline (TBS; 0.05 M Tris and 0.15 M sodium chloride, pH 7.4) was used to prevent non-specific binding (1 h at 37 °C) followed by incubation with a primary antibody raised against CDKN2A/p16^Ink4a^ (M-156, #sc-1207; Santa Cruz Biotechnology Inc., Heidelberg, Germany) at a dilution of 1:100 in DAKO antibody diluent (#S0809; DAKO, Ely, UK) at 4 °C overnight. After the incubation with primary antibody, sections were washed twice for five minutes in TBS and incubated with goat anti-rabbit HRP-conjugated secondary antibody (1:200; #P0448; DAKO, Ely, UK) in normal goat serum for 30 min at 37 °C. After washes in TBS, sections were stained with 3,3′-Diaminobenzidine (DAB) chromogen (#SK-4100; Vector Laboratories, Peterborough, UK) and the reaction was stopped by rinsing in water. Sections were counterstained with Harris Haematoxylin (Sigma-Aldrich, Dorset, UK), dehydrated, and mounted according to the manufacturer’s guidelines. Negative controls were processed utilizing an identical methodology and conditions. Histoscores were generated by double-blind scoring of p16^Ink4a^ staining for brain sections from both strains of rat at two time points (two months and 12 months old). 

### 2.3. Senescence-Associated β-Galactosidase (SA-β-gal) Staining

Briefly, slides were equilibrated to room temperature, rehydrated in PBS for 10 min, and incubated with two SA-β-gal solutions at pH 6 (for test sections) and pH 4 (positive control) for 48 h at 37 °C. Basic staining solutions used for test slides and positive controls were prepared (1 mg of X-Gal, 5 mM potassium ferricyanide, 5 mM potassium ferrocyanide, 2 mM magnesium chloride, 150 mM sodium chloride in PBS, and 40 mM citric acid/sodium phosphate buffer) and adjusted to pH 4 (positive control slides) and pH 6 (test slides). After incubation with staining solutions, slides were washed, counterstained with Nuclear Fast Red solution according to the manufacturer’s protocol, dehydrated, and mounted with DPX mounting medium (Sigma-Aldrich, UK). Senescent cells were stained blue. Histoscores were generated by double-blind scoring of SA-β-gal staining for both strains of rat, at two time points (two months and 12 months old). 

### 2.4. RNA Isolation and qPCR Analysis

Tissue specimens corresponding to the various brain regions (cerebellum, brain stem, cerebral cortex and basal ganglia) were dissected from the brain of AS and AS/AGU rat strains, snap-frozen in liquid nitrogen, and stored for further analysis. Total RNA was isolated using TRI^®^zol reagent according to the manufacturer’s instructions, followed by DNase treatment to remove genomic DNA contamination (as per the manufacturer’s instructions, Turbo DNase I, Promega, Southampton, UK). RNA quality and quantity was estimated spectrophotometrically (NanoDrop^®^2000, Thermo Fisher Wilmington, USA) and 500 ng of total RNA was subjected to reverse transcription using SuperScript II^®^ (Invitrogen, Paisley, UK). QPCR was performed using pre-designed TaqMan^®^ probes: Cdkn2A/p16 (Rn00580664_m1), Sirt5 (Rn01450559_ml), Sirt6 (Rn01408249_ml), Sirt7 (Rn01471420_ml), and Glyceraldehyde 3-phosphate dehydrogenase (GAPDH, Rn01775763_g1) as an endogenous control according to the manufacturer’s protocols. Each sample was run in triplicate and a standard deviation between cycle threshold (Ct) values of less than 0.25 was considered as acceptable for further analyses. Relative gene expression was calculated using the comparative threshold (Ct) method [30].

### 2.5. Statistics

All datasets were tested for normality and the unpaired *t*-test (two-tailed) was used to demonstrate differences between the means of experimental groups and between brain regions; *p* values are presented to three decimal places. A 95% CI (*p <* 0.05) was used throughout to determine significance, however, significance at the 99% CI (*p <* 0.01) and 99.9% CI levels (*p <* 0.001) are also denoted where appropriate.

## 3. Results

### 3.1. AS/AGU Rat Brains Exhibit Features Consistent with Accelerated Aging

To determine whether the phenotype of the AS/AGU mutant exhibited features of accelerated aging, two validated markers of cellular aging were evaluated. These comprised CDKN2A/p16^Ink4a^ analysed at both the transcriptional and translational levels and SA-β-gal staining.

**Figure 1:**
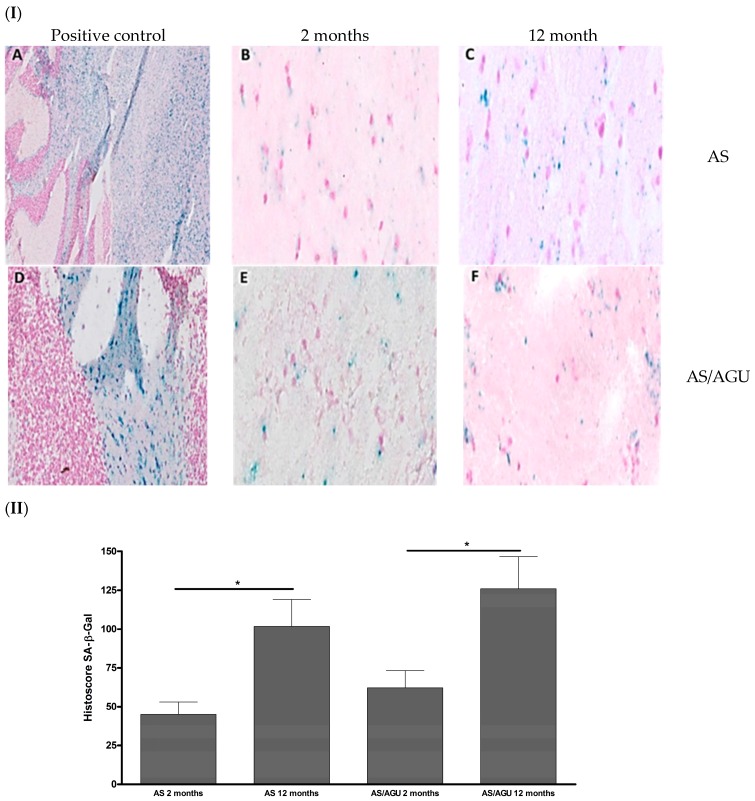
Senescence-associated β-galactosidase (SA-β-gal) in the sagittal sections of AS and AS/AGU rat stains in relation to age. (**I**) Immunohistochemical (SA-β-gal) activity. **A**: Twelve month AS pH4 (control). **B**: Two month AS pH6. **C**: Twelve month AS pH6. **D**: Two month AS/AGU pH4 (control). **E**: Two months-AS/AGU pH6. **F**: Twelve month AS/AGU pH6. Magnification 100×. (**II**) Total histoscore for SA-β-Gal stain (senescent cells stained in blue colour) in the brain sections of both rat strains at the two time points. Significance: * *p <* 0.05.

AS/AGU animals exhibited a significant age related increase in SA-β-gal staining (*p =* 0.012), with older animals (12 months) displaying more SA-β-gal than younger rats (two months). Similar increase in SA-β-gal was observed in the case of the AS rat strain (*p* = 0.014, Figure 1).

**Figure 2:**
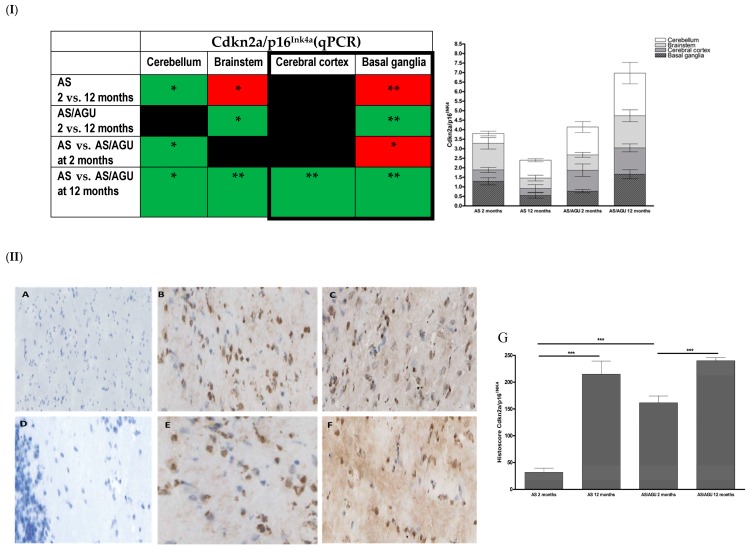
Age-related differences in Cdkn2a/p16^Ink4a^ expression in the brains of AS and AS/AGU rats. (**I**) Cdkn2a/p16 expression measured using qPCR. (**I**) Directionality of transcriptional changes denoted as colours (green-increase and red decrease in relative gene expression). Black colour denotes lack of significant changes in the gene expression. Regions of the brain linked to Parkinson’s disease are bolded for easier comparison). Stacked bar chart represents mean RQ Cdkn2a/p16^Ink4^ with SEM for each individual brain region in both rat strains at the two time points. Each bar represents the mean with SEM for an individual category. (**II**) Immunohistochemical staining and total histoscore for p16^Ink4a^ protein expression (brown precipitate) in para-sagittal brain sections collected from two month and 12 month old AS and AS/AGU rats. **A**: negative control (AS at 12 months old). **B**: Two month old AS. **C**: Twelve month old AS. **D**: Negative control (AS/AGU at two months old). **E**: Two month old AS/AGU. **F**: Twelve month old AS/AGU. **G**: Corresponding histoscores for p16^Ink4a^ expression in 12 month old AS and AS/AGU rats. Significance: * *p <* 0.05, ** *p <* 0.01, *** *p <* 0.001.

p16^Ink4a^ expression was significantly higher in older animals (12 months) compared to their younger counterparts in both AS (*p <* 0.001) and AS/AGU (*p <* 0.001) rat strains (Figure 2I). Interestingly, significantly higher levels of Cdkn2A/p16^Ink4a^ were noted in the AS/AGU rat strain aged two months compared to age-matched AS controls (*p <* 0.001, Figure 2II). The expression of Cdkn2A/p16^Ink4a^ in the AS/AGU rats at 12 months was significantly higher across all brain regions analysed in comparison to age-matched AS controls (cerebellum *p =* 0.032; brainstem *p =* 0.006; cerebral cortex *p =* 0.006; and basal ganglia *p =* 0.002).

The expression of the Cdkn2A/p16^Ink4a^ transcript was further analysed across brain regions to determine whether the increase in cellular senescence was segmental (differentially reflected in differing brain regions) (Figure 2I). Significantly higher Cdkn2A/p16^Ink4a^ expression was observed in the brainstem (*p =* 0.046) and basal ganglia (*p =* 0.006) regions of 12 months old AS/AGU rats compared to two month old animals. This was not observed in AS animals. 

The AS strain displayed an age related increase in Cdkn2A/p16^Ink4a^ expression within the cerebellum region, with a concomitant decrease within the brainstem (*p =* 0.030) and basal ganglia (*p =* 0.009) regions (Figure 2I). Interestingly, greater Cdkn2A/p16^Ink4a^ expression was also observed in the cerebellum of two month old AS/AGU rats compared to the AS controls while, conversely, within the basal ganglia decreased expression was noted (*p =* 0.032).

### 3.2. Age-Related Changes in Cell Stress in the Brain Are Reflected in Differing Expressions of Members of the Sirtuin Family

**Figure 3:**
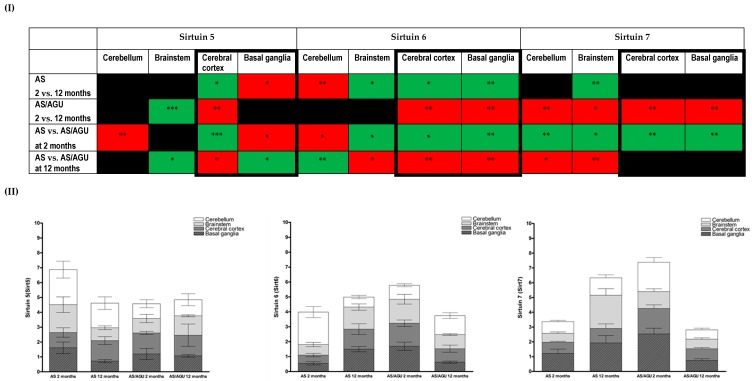
(**I**) A summary of Sirt5, Sirt6, and Sirt7 gene expression measured using qPCR across brain regions in AS and AS/AGU strains at two and 12 months. Green colour denotes an increase, while red denotes a decrease in relative gene expression. Significance: * *p <* 0.05, ** *p <* 0.01, *** *p <* 0.001. Black colour denotes the lack of significant changes in the gene expression. Regions of the brain linked to the Parkinson’s disease are bolded for easier comparison. (**II**) Stacked bar charts represent mean RQ sirtuin values with SEM for each individual brain region in both rat strains at the two time points. Each bar represents the mean with SEM for an individual category.

The expression of selected members of the sirtuin family across brain regions (cerebellum, brainstem, cerebral cortex, and basal ganglia) was further analysed in the AS and AS/AGU rats at the same two time points, to determine whether an increased senescence–associated phenotype could be related to cellular homeostasis in the context of biological aging. The data from these are summarized in Figure 3.

Significant changes were observed for transcriptional expression of Sirt5 in the cerebral cortex and basal ganglia regions between 12 month old AS rats and two month old animals. These are mirrored between strains, with additional changes observed within the cerebellum of the AS/AGU strain (Figure 3I,II). Additionally, the pattern of age-related Sirt5 relative expression across the cerebral cortex and basal ganglia was reversed in an inter-strain comparison. Interestingly, Sirt5 expression within the brainstem and cerebral cortex of old (12 months) in relation to young AS/AGU rats was similar to that observed when both strains are compared at 12 months. 

Age-related changes in Sirt6 transcriptional expression were also apparent. Significantly higher age related Sirt6 expression was noted in the brainstem, cerebral cortex, and basal ganglia (*p <* 0.01, Figure 3I,II), while a decrease was observed in the cerebellum in AS animals. AS/AGU animals displayed an inverted image of this, with decreased age-related expression in the cerebral cortex and basal ganglia. 

An inter-strain comparison showed the pattern of Sirt6 expression between AS and AS/AGU rats compared at the two time points, was reversed across brain regions when both strains were compared at two and 12 months, with three regions (brainstem, cerebral cortex, and basal ganglia) revealing similar changes in the directionality of expression while, within the cerebellum, Sirt6 expression was divergent (Figure 3I,II). 

A decrease in Sirt7 expression across all analysed brain regions was observed in AS/AGU rats at 12 months as compared to two months (*p <* 0.05), with similar changes for the cerebellum and brainstem seen when both strains were compared at 12 months (Figure 3I,II). Interestingly, the expression of Sirt7 is increased in AS/AGU rats at two months but this pattern is reversed, with Sirt7 being significantly lower, in the cerebellum and brainstem, when 12 month animals from both strains are compared (Figure 3I,II).

## 4. Discussion

This study has demonstrated that the AS/AGU rat strain presents with features of accelerated segmental aging in comparison to its parental AS strain, as a direct result of a single PKCγ point mutation. This animal model has provided an opportunity to analyse how the associated disruption of neurotransmitter release affects aging within the brain, by direct comparison of the AS/AGU mutant strain with its parental AS strain. The manifestation of this accelerated segmental aging is reflected in differential expression of established biomarkers of cellular senescence, namely CDKN2A/p16^Ink4a^ and SA-β-gal, alongside markers of cellular metabolic stress (Sirt5, Sirt6, and Sirt7). Our data clearly show differential accumulation of markers of cellular senescence in the brain. This indicates that an accelerated segmental aging phenotype in AS/AGU rats can be characterized by a significant increase in the expression of such markers, compared to the parental AS strain, even at an early life point. To our knowledge, this is the first demonstration of segmental aging in the mammalian brain. A similar phenomenon could be present in diseases associated with the accelerated ageing phenotype, including chronic kidney disease (CKD), obstructive pulmonary disease (COPD) or viral infections (HIV, HCV) [12,13,31,32,33,34].

SA-β-gal is widely used as a marker for senescent cells and appears to increase with age in a wide range of eukaryotic model organisms. Its accumulation reflects lysosomal elevation of β-galactosidase levels and an increase in lysosomal mass in response to cellular stressors [35,36,37]. Interestingly, p16^Ink4a^ and SA-β-Gal expression can be driven by β-amyloid in senescent astrocytes; however, the accumulation of SA-β-gal depends on pRB (retinoblastoma) functionality and is associated with the p21^WAF1^-mediated senescence pathway, independently of SA-β-gal status [38,39]. Others have stipulated that an overall increase in SA-β-gal expression in the aging brain may be associated with changes in ganglioside abundance, but ultimately this can also be linked to senescent neurons and can be considered as a useful surrogate marker of neurodegenerative diseases [40,41,42]. Similarly, up-regulation of p16^Ink4a^ is a consistent feature of cellular senescence and age-related morbidity in a range of in vitro and in vivo models. However, direct evidence supporting a role for p16^Ink4a^ in brain physiology is still deficient and is mostly supported by circumstantial evidence, including the involvement of p53 and Rb moieties in response to brain trauma, aetiology of neurodegenerative diseases or cancer [43,44,45,46,47,48,49,50]. 

As expected, increased expression of SA-β-gal was observed in older animals regardless of the strain. These data are consistent with increased cellular senescence in the brains of AS/AGU animals concomitant with the development of impaired locomotor function and shortened lifespan [51]. The data on SA-β-gal expression are particularly intriguing, as they indicate the potential for differing paracrine effects resulting from PKCγ mutation in AS/AGU rat strain and their association with segmental brain aging.

Although a significant increase was observed for Cdkn2a expression in older animals, regardless of strain, this was exaggerated in AS/AGU rats compared to age-matched controls, from as early as two months old. These data are indicative not only of an accelerated aging phenomenon in early life, but also of segmental aging across brain regions between both rat strains. Interestingly, the Cdkn2a expression patterns in the cerebellum and basal ganglia in young AS/AGU rats (two months) were similar to those observed in the older AS parental strain (12 months), with a further increase in expression of Cdkn2a across all brain regions in the AS/AGU strain at 12 months. Differential Cdkn2a expression in the brainstem region, which controls sleep cycle and autonomic respiratory and cardiac functions, suggest that this region may be more affected by stress in older AS/AGU rats as compared to the control AS strain.

The differences observed in cellular aging were also reflected in the expression of markers of metabolic stress inherent to the MTR theory [19]. The sirtuin markers used in this study represent members of a highly conserved family of proteins with functions common across the taxa. These have not been analysed previously in the context of Parkinson’s disease pathogenesis, in contrast to other sirtuin family members (Sirt1, Sirt2, and Sirt3). Those chosen for analysis in this study have been implicated in the regulation of aging processes, where they provide a unique link between cellular metabolism and cellular and genomic stress responses [19,20,52,53,54]. As the brain has the highest energy requirement in the body in order to maintain its functionality their activity can, therefore, be considered as reflective of the state of cellular metabolism within the organ, and of responses to cellular damage linked to the aging in the brain.

The changes in sirtuin expression levels observed in the current study are congruent with results from other studies. Typically, respective sirtuin expression decreases with age, with the exception of Sirt7 [22,55,56]. This reduction may be indicative of DNA damage accumulation and may be a precursor to apoptosis when damage is too great to repair. Overall, the expression of sirtuins appears to follow a pattern which suggests that expression in AS/AGU rats is increased early in life. Given the roles of three sirtuins (Sirt5, Sirt6, and Sirt7) in the prevention of oxidative stress, repair of DNA damage, and modulation of cellular metabolism, it is highly suggestive that at least one of these factors can be linked to the accelerated aging phenotype of the AS/AGU rats. 

In keeping with such a hypothesis, decreased expression of Sirt5 has been proposed as a risk factor for Parkinson’s disease (PD) in humans and as a marker for the decline in cognitive capacity. This is thought to be due to a metabolic shift occurring with increased age and morbidity and concomitant mitochondrial dysfunction [57,58]. Changes in Sirt6 expression have also been associated with an increase in genome instability, and linked to changes in cellular metabolism, inclusive of a reduced capacity to respond to stress. In keeping with this, and congruent with the postulates of the MTR, Sirt7 has been demonstrated to be critical for ribosomal stability, inherent in the regulation of RNA metabolism and protein synthesis. Changes in the expression of this moiety have also been linked directly to changes in metabolic homeostasis. 

How mutation of PKCγ leads to segmental accelerated aging remains to be proven, but may reflect a combination of the physiological and cellular stresses resulting from dopaminergic neuronal loss and associated paracrine signalling changes across the brain over the animals life course.. 

An increase in the Sirt5 expression at early age time points in AS/AGU rats is indicative of greater extant physiological stress in the mutant and reflects the brain’s compensatory responses to the metabolic dysregulation and mitochondrial stress. This has previously been proposed as a major mechanism for Parkinson disease aetiology [59]. This is of particular interest, as recent studies have revealed the importance of Sirt5 in the regulation of proteins involved in essential metabolic pathways, including fatty-acid oxidation, oxidative phosphorylation, energetic flux through glycolysis, and ketone body production [60,61]. Moreover, it has been also demonstrated that Sirt5 can be co-localized with cytochrome C controlling its acetylation levels, thus, Sirt5 would be critical for oxidative metabolism in response to stress, as well as regulating the initiation of apoptosis [62]. The importance of Sirt5 in the preservation of mitochondrial function has been highlighted in an MPTP-induced model of PD, where the absence of Sirt5 expression exacerbated the nigrostriatal dopaminergic degeneration induced by MPTP [23]. Other studies have also revealed that Sirt5 can be considered a major component of metabolic adaptivity, via a role in the regulation of detoxification processes (i.e., removal of excess ammonia produced when amino acids are used as a source of energy during fasting, caloric restriction, or high-protein diet) and in the regulation of autophagy and mitophagy [63,64]. 

Such a response to stress, however, cannot be maintained in AS/AGU rats and results in an increase in cellular senescence, reflected in Cdkn2a and SA-β-gal expression. This may lead to a ‘’critical point’’ in the mutants physiology, resulting the accelerated aging phenotype apparent at an earlier age time point (two months), with a similar presentation to the older rats originating from the parental strain. Similarly, changes in Sirt6 and Sirt7 expression may reflect disruption of cellular homeostasis (e.g., glucose uptake and glycolysis) and genomic instability. Changes in the expression of Sirt6 in the younger AS/AGU rats may be related to lower glucose uptake leading, consequently, to lower body weight [7]. However, this is in contrast to other mouse models with neural deletion of Sirt6, which leads to early growth retardation and obesity [24], while overexpression of Sirt6 in different models extends lifespan and protects against diet-induced obesity [65,66]. The changes observed in Sirt6 expression may be a composite of its roles in metabolic regulation and DNA damage responses. While it may initially function to prevent the accumulation of DNA damage at an early age, it gradually fails to enable regulation of metabolic homeostasis in the AS/AGU strain, which is then reflected in the progressive development of the mutant phenotype.
